# Non-Destructive Measurement of Acetic Acid and Its Distribution in a Photovoltaic Module during Damp Heat Testing Using pH-Sensitive Fluorescent Dye Sensors

**DOI:** 10.3390/s22072520

**Published:** 2022-03-25

**Authors:** Hideaki Nagasaki, Takashi Asaka, Kentaro Iwami, Norihiro Umeda, Chizuko Yamamoto, Yukiko Hara, Atsushi Masuda

**Affiliations:** 1 Department of Mechanical System Engineering, Tokyo University of Agriculture and Technology, Koganei 184-8588, Japan; nagasaki.hideaki@gmail.com (H.N.); moon.ride.360storm@gmail.com (T.A.); umeda@cc.tuat.ac.jp (N.U.); 2 Research Center for Photovoltaics, National Institute of Advanced Industrial Science and Technology, Tsukuba 305-8568, Japan; chizuko.yamamoto.pv@gmail.com (C.Y.); hara.yukiko@nims.go.jp (Y.H.); a-masuda@eng.niigata-u.ac.jp (A.M.); 3 Graduate School of Science and Technology, Niigata University, Niigata 950-2181, Japan

**Keywords:** photovoltaic, acetic acid, pH measurement, SNARF-4F

## Abstract

An optical pH sensor that enables the non-destructive measurement of acetic acid and its distribution in a photovoltaic module during damp heat (DH) testing is reported. The sensor was fabricated by impregnating a solution of a pH-sensitive fluorescent dye into a fluororesin membrane filter, which was then dried. While conducting the DH test, fluorescence spectra from 20 pH sensors were periodically recorded and converted into pH values using a predetermined calibration curve. As a result, we succeeded in measuring changes in pH with a DH test time of up to 2000 h, and it was possible to obtain information on the pH distribution in the module. We also confirmed no change in pH in a module with a silicone encapsulant free from acetic acid, and revealed that the sensor that we developed does not respond to moisture and heat, but only to acetic acid.

## 1. Introduction

Although photovoltaic (PV) energy is significant renewable and its use is spreading rapidly, high generation costs represent an obstacle to further expansion [1,2]. It has been proposed that the lifetimes of PV modules should be determined by high-temperature and high-humidity (damp heat: DH) tests based on IEC61215 standard [3], which are forms of environmental acceleration tests [4,5]. Recently, deterioration in the performance of a PV module was reported because ethylene-vinyl acetate copolymer resin (EVA), which was used as an encapsulant, was hydrolyzed by moisture that penetrated the module to generate acetic acid, which corroded the electrode of the solar cell [6,7,8,9,10,11,12,13]. Acetic acid can be generated through several paths including the Norrish reaction due to thermal or photothermal stimulation, ref. [11] or hydrolysis [8] from EVA. Furthermore, acetic acid attacks glass layer in Ag electrode on a PV cell, resulting in increase the series resistance [14,15,16,17,18]. To prolong the lifetime of a PV module, it is necessary to detect acetic acid in the module and to reveal the mechanism by which it is generated [19,20,21,22,23,24]. It should be noted that acetic acid is the dominant product, with a small amount of formic acid also produced. The amount of formic acid relative to the amount of acetic acid is about 1/10 in the field test [25] and 1/100 in the accelerated test [26].

Currently, although the state of degradation of PV modules is determined by measurements of the acetic acid concentration using ion chromatography analysis, this method is destructive when used to measure the acetic acid concentration in a PV module [14]. Therefore, a method for the non-destructive continuous monitoring of the deterioration of EVA is required. Although the use of Raman spectroscopy, which is a non-destructive detection method, has been proposed [5], the apparatus involved is expensive. Moreover, because the fluorescence background of EVA is more intense than that for Raman signals, the accuracy of measurements at the initial stage of DH testing is relatively low. Although the authors have been working on the non-contact temporal measurement of the acetic acid reaction amount using the reflectance change due to corrosion of tin thin film, the quantification of the concentration was not good [27,28].

Therefore, we devised a pH sensor with a thickness of about 0.1 mm, which contains a pH-sensitive fluorescent dye on a porous membrane [29,30], and clarified its pH characteristics and related problems. In this study, we aimed to measure temporal and spatial changes of pH values in a PV module by enclosing this sensor in the PV module and subjecting it to a DH test. Performance limitation of the pH sensor is also discussed.

## 2. Materials and Methods

### 2.1. Calibration between pH and Fluorescence Intensity Ratio

The acetate ion concentration can be estimated if the pH is known. In this study, the pH-sensitive fluorescent dye carboxy SNARF-4F (Invitrogen™, Thermo Fisher Scientific Inc., Waltham, MA, USA), which is a fluorescent pH indicator used in biological sciences, was used for the non-destructive measurement of pH [31]. SNARF-4F has two fluorescence peak wavelengths, namely, 587 and 650 nm, and its fluorescence intensity ratio (FIR), which is defined by Equation (1), changes reversibly depending on the pH [32]:

(1) $$FIR=I_{587}/I_{650}$$

where $I_{650}$ and $I_{587}$ are the fluorescence peak intensities at fluorescence wavelengths of 650 and 587 nm, respectively. Even if the fluorescent dye is dropped onto a glass substrate and dried to form a thin film, the sensitivity of the pH response may be relatively low because the amount of moisture that penetrates into the PV module is very small. Therefore, in order to extend the area of contact between moisture and the fluorescent dye, a sensor substrate for carrying the dye was prepared using a polytetrafluoroethylene fluororesin membrane filter with a pore size of 0.2 μm (Omnipore®, Merck Group, Burlington, MA, USA) [30]. Here the aqueous solution of the dye is based on ultrapure water, and the pH is not adjusted by the addition of basic substances.

A calibration curve of pH values to the FIR value of the manufactured sensor is necessary. For this purpose, the following calibration experiment was carried out.

1. First, a Britton–Robinson (BR) buffer solution was prepared so that the pH value did not significantly change when it was dripped onto the sensor [33]. For this purpose, phosphoric acid, boric acid, and acetic acid were prepared with a concentration of 0.04 M and diluted in a measuring cylinder to a total volume of 500 mL with ultrapure water.
2. Then, 0.2 M NaOH was injected into the BR buffer, and the amount of NaOH that was injected was adjusted to obtain a predetermined pH value while the pH was measured using a calibrated pH meter (SevenEasy pH, Mettler–Toledo International Inc., Greifensee, Switzerland). The pH range after adjustment was 2–11.
3. Finally, 20 μL pH-adjusted BR buffer was added to the prepared sensor substrate, and the fluorescence intensity spectrum and FIR were measured in the wet state with a spectrometer (QE65000, Ocean Insight Inc., Orlando, FL, USA). The intensity of the fluorescence excitation laser was 100 μW, and the integration time of the spectroscope was 10 s. The FIR value was calculated using Equation (1). Please note that every time a spectroscopic measurement was completed, the sensor substrate was replaced.

**Figure 1:**
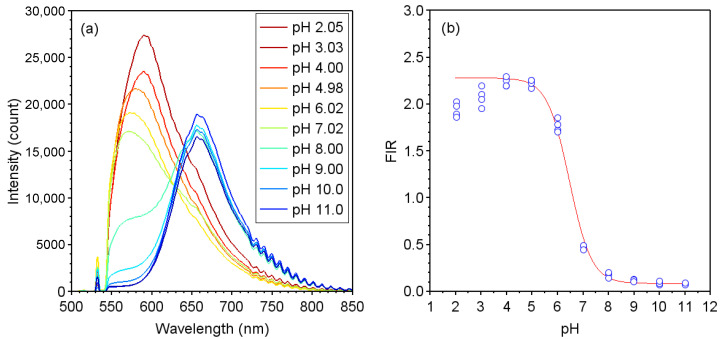
(**a**) Fluorescence spectra of pH sensor with SNARF-4F impregnated in the membrane filter; (**b**) FIR values calculated from the fluorescence spectra. The solid line is the fitting curve obtained using Equation (2).

Figure 1a shows the fluorescence spectrum of the sensor at each pH value calibrated by the pH meter. When the pH became acidic, the fluorescence wavelength peak shifted to 587 nm from 650 nm. Figure 1b shows the results of calculations of the FIR, which were obtained from the fluorescence spectrum and are plotted as a function of the pH. The solid line in the figure represents the result of the calibration curve expressed by Equation (2) [34]:

(2) $$FIR=\frac{FIR_{\min}\times G+FIR_{\max}\times 10^{pK_{a}-\mathrm{pH}}}{G+10^{pK_{a}-\mathrm{pH}}}$$

Here, p*K*_*a*_ represents the acid dissociation constant of SNARF-4F. $FIR_{\max}$ and $FIR_{\min}$ refer to the maximum and minimum values within the measurement range of SNARF-4F, respectively, *G* is the fitting parameter. The calibration curve is obtained using Equation (2) fitting to the experimental data points with the parameters of $FIR_{\max}$, $FIR_{\min}$, and *G*, and used to determine the pH value from the FIR. The values of each parameter are shown in Table 1. In addition, from Figure 1b, it was found that the pH range measurable with this sensor is approximately pH 4–8, in which the FIR maintains a monotonical increase with respect to decreasing pH. At the range below pH 4, $I_{650}$ reverses to increase, dragged by the increase in $I_{587}$, resulting in a decrease in FIR.

### 2.2. Experimental Procedure

**Figure 2:**
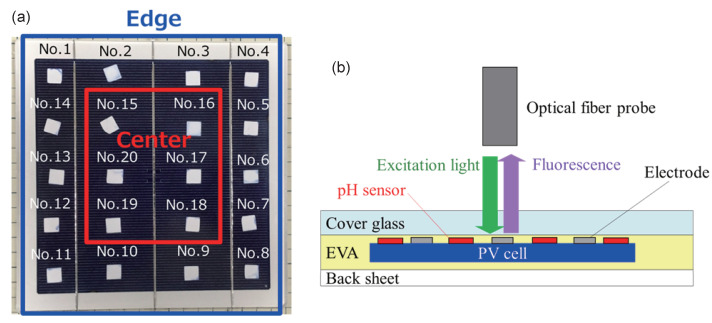
(**a**) Photograph of numbered pH sensors equally spaced on the PV module; (**b**) cross-sectional schematic diagram of the PV module and the method used for recording fluorescence spectra.

A sheet of fabricated pH sensors with a size of 18 × 18 mm^2^ was laminated in a PV module with a module size of 180 × 180 mm^2^ and a cell size of 156 × 156 mm^2^, which comprised a multicrystalline Si PV cell with an Ag-based grid. As shown in Figure 2a, the 20 sensors were arranged so as to be almost equally spaced on the upper surface of the PV cell. Here, the sensors numbered from No. 1 to No. 14 around the edge of the module are referred to as “edge”, and the sensors numbered from No. 15 to No. 20 inside the module are referred to as “center”. In addition, as shown in the cross-sectional view of the PV module in Figure 2b, the components of the PV module were a cover glass, EVA sheets used as encapsulants in front of and behind the PV cell, and a back sheet composed of polyvinyl fluoride:PVF 38 μm/polyethylene terephthalate:PET 250 μm/PVF 38 μm. The lamination of the module was conducted at 135 °C for 15 min. Please note that the edges surrounding the PV module were not processed with a sealant such as silicone or a gasket.

Measurements of fluorescence were carried out by placing a bundled optical fiber probe (R400-VIS-NIR, Ocean Insight Inc., Orlando, FL, USA) by the upper surface of the cover glass, as shown in Figure 2b, and irradiating the module using an excitation light source, namely, a yttrium-aluminum-garnet laser with a wavelength of 532 nm (intensity 100 μW). Fluorescence spectra were recorded with a spectrometer QE65000 by detecting fluorescence emitted from the pH sensor via the same probe, and the pH value was calculated from the FIR value using Equation (2).

The laminated PV module containing the pH sensor was installed in an environmental test chamber and exposed to a DH test environment (85 °C, 85% relative humidity). During the DH test, FIR values were determined from fluorescence spectroscopic measurements of the sensor by removing the module from the test chamber at certain time intervals, and the corresponding pH values were calculated. For comparison, pH measurement experiments were also conducted on a PV module with silicone resin as an encapsulant, which has been reported not to generate acetic acid [35].

## 3. Results

### 3.1. I−V Characteristics of PV Modules

**Figure 3:**
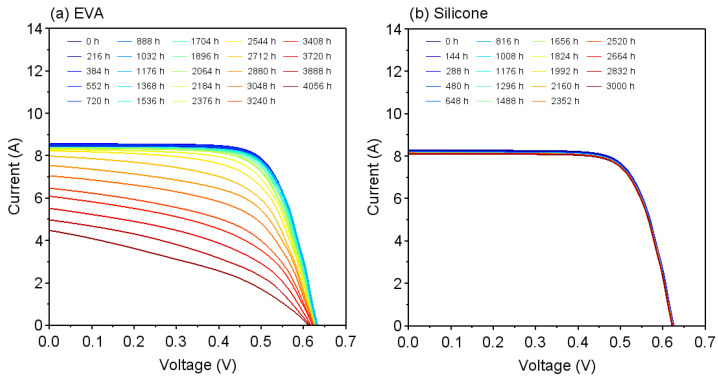
I−V characteristics during 85 °C, 85%RH DH test for modules with (**a**) EVA and (**b**) silicone encapsulants.

Figure 3 shows the current-voltage (I−V) characteristics of the PV modules as a function of the DH test time. Figure 3a,b show the results of measurements for the modules with EVA and silicone encapsulants, respectively. From these figures, it can be seen that in the module containing EVA, as shown in Figure 3a, remarkable decreases in the fill factor and short-circuit current, which are indices of the degradation of PV modules [36], were observed at DH test times of greater than 3000 h. This phenomenon is suggested to be caused by corrosion of the electrode due to the production of acetic acid within the PV module, as previously reported [14]. On the other hand, in the module containing silicone, as shown in Figure 3b, there was no significant deterioration in the I−V characteristics, which indicates that no acetic acid was generated in the module containing silicone.

### 3.2. Changes in FIR and pH Values

**Figure 4:**
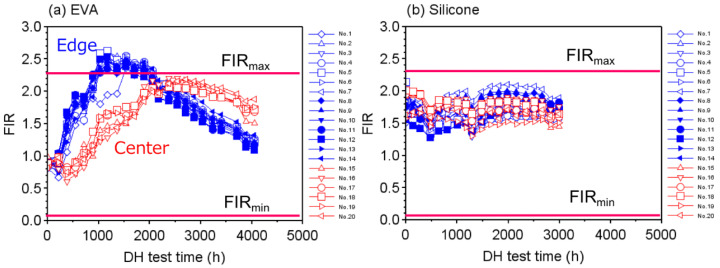
Temporal response of FIR values measured by the pH sensors as a function of the DH test time for modules with (**a**) EVA and (**b**) silicone encapsulants. $FIR_{\max}$ and $FIR_{\min}$ correspond to the values shown in Table 1.

The characteristics of the FIR values measured by the pH sensors were recorded for the respective modules with each encapsulant during the DH test. The results are shown in Figure 4. In this figure, $FIR_{\max}$ and $FIR_{\min}$ indicate the calibration parameters shown in Table 1. In the module with EVA, as shown in Figure 4a, the FIR for all the sensors in edge positions started to rise from a value of about 0.9 when the DH test time *t* was about 200 h. Although the FIR values increased almost proportionally with the value of *t* up to 1000 h, they became stable from t=1000 h to 2000 h and then decreased when *t* was around 2000 h. As can be seen from the calibration curve in Figure 1b, this may be assumed to have occurred because the acetic acid concentration had increased beyond the measurement range of SNARF-4F. The FIR values for the sensors in the center region started to increase at t=600 h, became stable at t=2000 h, and then decreased at t=3000 h. The reason the FIR values reached a maximum and then decreased is that it is saturated around pH 5 to 4 and decreases at pH 4.0 or lower as shown in Figure 1b.

On the other hand, in the case of the module with the silicone encapsulant, as shown in Figure 4b, the FIR values for both the edge and the center sensors were almost constant with no significant fluctuations, although there were differences in the initial values, which might have been due to variations in the FIR value of the sensor during fabrication. The results in Figure 4b indicate that no acidic substances were generated in the module.

**Figure 5:**
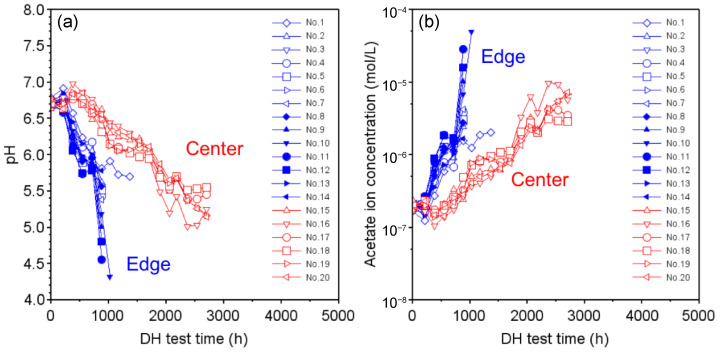
(**a**) Temporal response of pH values calculated using the calibration curve from FIR values measured by the edge and center sensors; (**b**) acetate ion concentrations calculated using the pH values.

Figure 5a shows the results of replotting the pH values calculated from the FIR values as a function of the DH test time using the calibration curve obtained via Equation (2) for the module with EVA encapsulant. As shown in Figure 4, the FIR values used for the calculation of pH values are limited to the range between the initial value at t=0 and the maximum value within the measurement range of SNARF-4F. For the edge sensors, as shown in Figure 5a, the pH value of 6.6 at the beginning of the DH test rapidly decreased to 4.5 by around t=1000 h. On the other hand, the pH value for the center sensors began to decrease at around t=600 h and decreased linearly to a pH of 5.0 up to t=2500 h.

Using the pH characteristics shown in Figure 5a, the acetate ion concentration was calculated, which represents a formula for the definition of pH, and the results are plotted in Figure 5b.

From this, it can be seen that the acetate ion concentration of $2\times 10^{-7}$ mol/L in the initial stage of the DH test increased more than 100-fold by around t=1000 h in the edge part of the module. On the other hand, in the central part, the concentration increased by a factor of about 70 by about t=2500 h.

### 3.3. Ph Mapping

**Figure 6:**
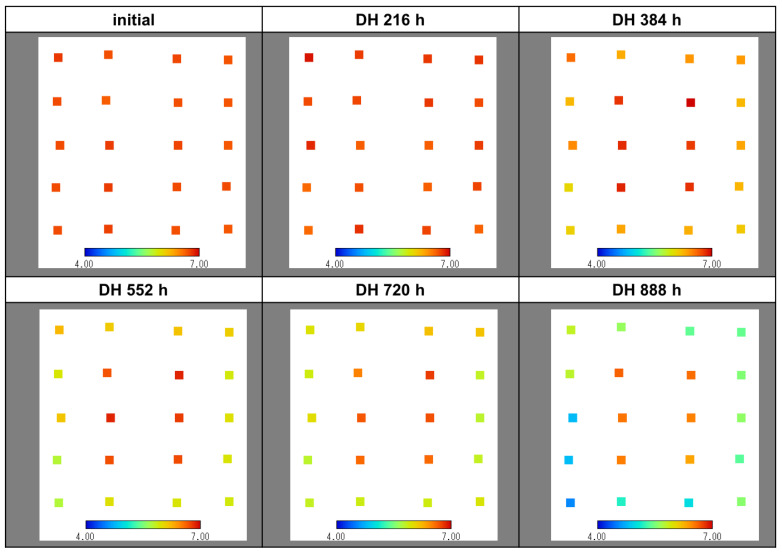
Changes in pH distribution within the PV module surface with respect to the DH test time.

As shown in Figure 2a, it was possible to simultaneously measure changes in pH at 20 points on the PV module surface. Therefore, the extent of the generation of acetic acid was confirmed by mapping the pH distribution within the PV module surface as a function of the DH test time, as shown in Figure 6. As seen in Figure 6, the pH remained almost constant at 7 until a DH test time of t=216 h, whereas at t=384 h the pH of the edge portion began to decrease. It was found that the acidity of the edge portion further increased at t=720 h and that the pH of the edge portion reached 4 before t=900 h. In particular, the increase in acidity in the lower left part of the module was faster than in other parts of the edge. This phenomenon seems to show the possibility that the degree of crosslinking of EVA varied due to the nonuniformity of lamination temperature during the fabrication of the PV module.

## 4. Discussion

Measured results are analyzed using two-dimensional reaction-diffusion equations as follows:

(3) $$\left\{\begin{array}{cc} \frac{\partial [H_{2}O]}{\partial t} & =\lambda_{w}\left(\frac{\partial^{2}\left[H_{2}O\right]}{\partial x^{2}}+\frac{\partial^{2}\left[H_{2}O\right]}{\partial y^{2}}\right)-a\exp\left(-\frac{E_{a}}{kT}\right)\left[H_{2}O\right]\\ \frac{\partial [CH_{3}COOH]}{\partial t} & =\lambda_{aa}\left(\frac{\partial^{2}\left[CH_{3}COOH\right]}{\partial x^{2}}+\frac{\partial^{2}\left[CH_{3}COOH\right]}{\partial y^{2}}\right)+a\exp\left(-\frac{E_{a}}{kT}\right)\left[H_{2}O\right] \end{array}\right.$$

**Figure 7:**
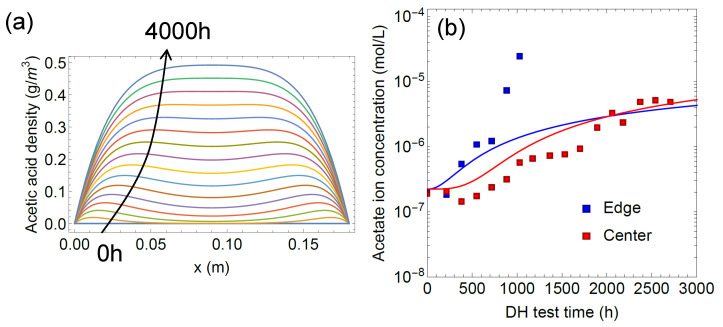
(**a**) Spatial distribution of acetic acid density. Each plot corresponds to the time from 0 to 4000 h for 250 h step. (**b**) Time variation of acetate ion concentration at the edge and center. Curves and points represent solution of Equation (3) and average of experimental results, respectively.

Where $\lambda_{w}$ and $\lambda_{aa}$ are diffusion constants of water and acetic acid in EVA [8,20], *k* is the Boltzmann constant, *T* is the temperature, $E_{a}$ is the Arrhenius parameter of hydrolysis reaction, ref. [8] and *a* is the fitting parameter related to the density of acetic acid group in EVA. The initial condition of both [H_2_O] and [CH_3_COOH] were zero. The boundary conditions of [H_2_O] and [CH_3_COOH] were the saturation density of water in EVA [20] and zero, respectively. Equation (3) is solved on a 180-mm-square two-dimensional space. Figure 7 shows the spatial distribution of acetic acid density (a) and time variation of acetate ion concentration at the edge and center (b). Curves and points in Figure 7b represent solution of Equation (3) and the average of experimental results, respectively. The consistent trend of acetic acid concentration increasing first at the edge and later at the center suggests that the measurements obtained by the SNARF-4F sensors reflect the change in acetic acid concentration due to the reaction-diffusion phenomenon.

As shown in the I−V characteristics of Figure 3a, degradation of power generation performance was observed with DH test time longer than 3000 h. In contrast, as shown in Figure 6, a decrease in pH during the DH test was observed at 384 h on edge and 720 h on the center. This result shows that SNARF-4F sensors can detect acetic acid much earlier than the degradation of electrical characteristics begins. This indicates the usefulness of the sensor for the early-stage detection of signs of degradation.

As a future subject of research, it is necessary to develop a fluorescent dye capable of detecting a pH of 4 or less and clarifying the conditions for the production of acetic acid in the module during DH testing for over 2000 h. It is also conceivable that the behavior of acetic acid in the module can be explained by the parametric fitting of the diffusion equation, which can quantitatively explain the diffusion of moisture into the PV module and the generation of acetic acid.

Another approach will be the use of micro pH sensors, including silver chloride electrodes [37] and ion-sensitive field effect transistors [38,39], integrated with wireless power supply or energy harvesting devices. For this approach, sensors for harsh testing environments, for example combination with DH and ultraviolet exposure or DH and high electric potential, will be required.

## 5. Conclusions

We attempted the non-destructive detection of acetic acid in a PV module during a DH test using a pH-sensitive fluorescent dye sensor. From the results, we demonstrated that it is possible to measure initial changes at DH test times of the order of 1000–2000 h and to measure the pH distribution in the plane of the module. In a module containing EVA, the rate of change in pH and the rate of increase in the acetate ion concentration were different in the edge and central parts of the module. On the other hand, from the results for a module containing silicone, it was shown that the values indicated by the sensor did not change upon mere exposure to moisture or heat in the DH test. It was confirmed that the SNARF-4F sensor that we developed can measure the initial behavior of the generation of acetic acid in the module containing EVA as an encapsulant.

## Figures and Tables

**Table 1:** Parameters for the calibration curve shown in Figure 1b obtained using Equation (2).

**p** ***K***_***a***_	${FIR}_{\min}$	${FIR}_{\max}$	***G***
6.4	0.083	2.278	0.851

## Data Availability

Not applicable.

## References

[1] Green M.A., Emery K., Hishikawa Y., Warta W., Dunlop E.D. (2012). Solar cell efficiency tables (version 39). Prog. Photovolt. Res. Appl..

[2] King D.L., Quintana M.A., Kratochvil J.A., Ellibee D.E., Hansen B.R. (2000). Photovoltaic module performance and durability following long-term field exposure. Prog. Photovolt. Res. Appl..

[3] International Electrotechnical Commission (2005). IEC 61215 Ed. 2.0.

[4] Wohlgemuth J.H., Kempe M.D. Equating damp heat testing with field failures of PV modules. Proceedings of the 2013 IEEE 39th Photovoltaic Specialists Conference (PVSC).

[5] Peike C., Kaltenbach T., Weiβ K.-A., Koehl M. (2011). Non-destructive degradation analysis of encapsulants in PV modules by Raman spectroscopy. Sol. Energy Mater. Sol. Cells.

[6] Masuda A., Yamamoto C., Hara Y., Ionue M., Uchiyama N., Doi T. Degradation Mechanism of Photovoltaic Modules by Extended Damp-Heat or Thermal-Cycle Test. Proceedings of the 28th European Photovoltaic Solar Energy Conference and Exhibition.

[7] Agroui K., Collins G. (2003). Characterisation of EVA encapsulant material by thermally stimulated current technique. Sol. Energy Mater. Sol. Cells.

[8] Kempe M.D., Jorgensen G.J., Terwilliger K.M., McMahon T.J., Kennedy C.E., Borek T.T. (2007). Acetic acid production and glass transition concerns with ethylene-vinyl acetate used in photovoltaic devices. Sol. Energy Mater. Sol. Cells.

[9] Peike C., Hoffmann S., Hülsmann P., Thaidigsmann B., Weiβ K.-A., Koehl M., Bentz P. (2013). Origin of damp-heat induced cell degradation. Sol. Energy Mater. Sol. Cells.

[10] Ketola B., Norris A. Degradation Mechanism Investigation of Extended Damp Heat Aged PV Modules. Proceedings of the 26th European Photovoltaic Solar Energy Conference and Exhibition.

[11] Czanderna A.W., Pern F.J. (1996). Encapsulation of PV modules using ethylene vinyl acetate copolymer as a pottant: A critical review. Sol. Energy Mater. Sol. Cells.

[12] Allen N.S., Edge M., Rodriguez M., Liauw C.M., Fontan E. (2000). Aspects of the thermal oxidation, yellowing and stabilisation of ethylene vinyl acetate copolymer. Polym. Degrad. Stab..

[13] Poulek V., Šafránková J., Černá L., Libra M., Beránek V., Finsterle T., Hrzina P. (2021). PV Panel and PV Inverter Damages Caused by Combination of Edge Delamination, Water Penetration, and High String Voltage in Moderate Climate. IEEE J. Photovolt..

[14] Masuda A., Uchiyama N., Hara Y. (2015). Degradation by acetic acid for crystalline Si photovoltaic modules. Jpn. J. Appl. Phys..

[15] Wendling M.D., Mondon A., Kraft A., Bartsch J., Glatthaar M., Glunz S.W. (2012). Analysis of chemical stability of printing pastes in electrochemical plating solutions. Energy Procedia.

[16] Olweya S., Kalio A., Kraft A., Deront E., Filipovic A., Bartsch J., Glatthaar M. (2013). Fine-line Silver Pastes for Seed Layer Screen Printing with Varied Glass Content. Energy Procedia.

[17] Kraft A., Wolf C., Lorenz A., Bartsch J., Glatthaar M., Glunz S.W. (2014). Long Term Stability Analysis of Copper Front Side Metallization for Silicon Solar Cells. Energy Procedia.

[18] Kraft A., Labusch L., Ensslen T., Durr I., Bartsch J., Glatthaar M., Glunz S., Reinecke H. (2015). Investigation of Acetic Acid Corrosion Impact on Printed Solar Cell Contacts. IEEE J. Photovolt..

[19] Jorgensen G.J., Terwilliger K.M., DelCueto J.A., Glick S.H., Kempe M.D., Pankow J.W., Pern F.J., McMahon T.J. (2006). Moisture transport, adhesion, and corrosion protection of PV module packaging materials. Sol. Energy Mater. Sol. Cells.

[20] Kempe M.D. (2006). Modeling of rates of moisture ingress into photovoltaic modules. Sol. Energy Mater. Sol. Cells.

[21] Miller D.C., Muller M.T., Kempe M.D., Araki K., Kennedy C.E., Kurtz S.R. (2012). Durability of polymeric encapsulation materials for concentrating photovoltaic systems. Prog. Photovolt. Res. Appl..

[22] Skoczek A., Sample T., Dunlop E.D. (2009). The results of performance measurements of field-aged crystalline silicon photovoltaic modules. Prog. Photovolt. Res. Appl..

[23] Wang E., Yang H.E., Yen J., Chi S., Wang C. (2013). Failure Modes Evaluation of PV Module via Materials Degradation Approach. Energy Procedia.

[24] Miyashita M., Masuda A. Correlation between Moisture Ingress and Performance in Photovoltaic Modules. Proceedings of the 28th European Photovoltaic Solar Energy Conference and Exhibition.

[25] Matsuda K., Watanabe T., Sakaguchi K., Yoshikawa M., Doi T., Masuda A. (2012). Microscopic Degradation Mechanisms in Silicon Photovoltaic Module under Long-Term Environmental Exposure. Jpn. J. Appl. Phys..

[26] Masuda A. (2013). (National Institute of Advanced Industrial Science and Technology, Tsukuba, Ibaraki, Japan).

[27] Hamaoka R., Iwami K., Itayama T., Nagasaki H., Takemoto S., Yamamoto C., Hara Y., Masuda A., Umeda N. (2018). Detection of acetic acid produced in photovoltaic modules based on tin film corrosion during damp heat test. Jpn. J. Appl. Phys..

[28] Asano S., Hamaoka R., Jonai S., Hara Y., Masuda A., Umeda N., Iwami K. (2022). Acetic acid detection in photovoltaic modules during ultraviolet irradiation and damp-heat combined tests. Jpn. J. Appl. Phys..

[29] Asaka T., Iwami K., Taguchi A., Umeda N., Masuda A. (2014). Detection of acid moisture in photovoltaic modules using a dual wavelength pH-sensitive fluorescent dye. Jpn. J. Appl. Phys..

[30] Asaka T., Itayama T., Nagasaki H., Iwami K., Yamamoto C., Hara Y., Masuda A., Umeda N. (2015). Development of a pH sensor based on a nanostructured filter adding pH-sensitive fluorescent dye for detecting acetic acid in photovoltaic modules. Jpn. J. Appl. Phys..

[31] Liu J., Diwu Z., Leung W.-Y. (2001). Synthesis and photophysical properties of new fluorinated benzo[c]xanthene dyes as intracellular pH indicators. Bioorganic Med. Chem. Lett..

[32] Kanazashi Y., Li Y., Onojima T., Iwami K., Ohta Y., Umeda N. (2012). pH Measurement Using Dual-Wavelength Fluorescent Ratio by Two-Photon Excitation for Mitochondrial Activity. Jpn. J. Appl. Phys..

[33] Mongay C., Cerda V. (1974). VI/A Britton-Robinson buffer of known ionic strength. Ann. Chim..

[34] Salerno M., Ajimo J.J., Dudley J.A., Binzel K., Urayama P. (2007). Characterization of dual-wavelength seminaphthofluorescein and seminapthorhodafluor dyes for pH sensing under high hydrostatic pressures. Anal. Biochem..

[35] Ketola B., McIntosh K.R., Norris A., Tomalia M.K. Silicones for Photovoltaic Encapsulation. Proceedings of the 23rd European Photovoltaic Solar Energy Conference and Exhibition.

[36] Tanahashi T., Sakamoto N., Shibata H., Masuda A. Electrical detection of gap formation underneath finger electrodes on c-Si PV cells exposed to acetic acid vapor under hygrothermal conditions. Proceedings of the 2016 IEEE 43rd Photovoltaic Specialists Conference (PVSC).

[37] Huang W.-D., Deb S., Seo Y.-S., Rao S., Chiao M., Chiao J.C. (2012). A passive radio-frequency pH-sensing tag for wireless food-quality monitoring. IEEE Sens. J..

[38] Jimenez-Jorquera C., Orozco J., Baldi A. (2010). ISFET based microsensors for environmental monitoring. Sensors.

[39] Xu F., Yan G., Wang Z., Jiang P. (2015). Continuous accurate pH measurements of human GI tract using a digital pH-ISFET sensor inside a wireless capsule. Measurement.

